# Supplementary material for: Tead1a Initiates Transcriptional Priming Through the TEAD1a/YAP‐Notch1‐Spi1/Cebpα Axis to Promote Neutrophil Fate
Source: Adv Sci (Weinh). 2025 Aug 28;12(41):e05441. doi: 10.1002/advs.202505441 (PMC12591127; doi:10.1002/advs.202505441)
Supplement: Supplementary file 4 — Supplementary Dataset [file ADVS-12-e05441-s004.zip › 3-8.zip!/3-8/0904/0904/05.annot/dimplot_celltype_cluster.pdf]

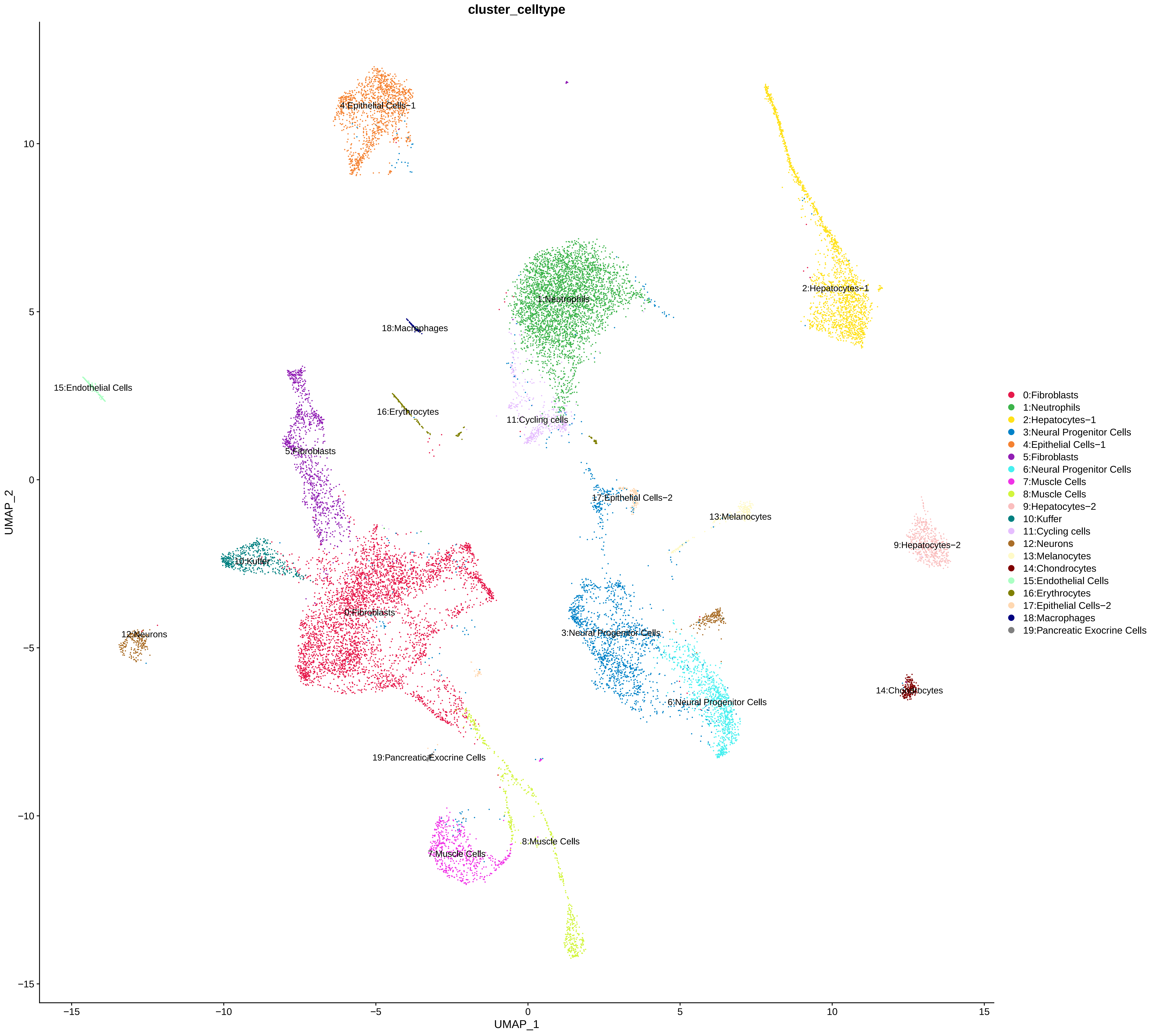

cluster_celltype
4:Epithelial Cells−1
2:Hepatocytes−1
1:Neutrophils
18:Macrophages
15:Endothelial Cells
16:Erythrocytes
11:Cycling cells
5:Fibroblasts
17:Epithelial Cells−2
13:Melanocytes
9:Hepatocytes−2
10:Kuffer
0:Fibroblasts
12:Neurons
3:Neural Progenitor Cells
14:Chondrocytes
6:Neural Progenitor Cells
19:Pancreatic Exocrine Cells
8:Muscle Cells
7:Muscle Cells
UMAP_1
UMAP_2
10
5
0
−5
−10
−15
−15
−10
−5
0
5
10
15
0:Fibroblasts
1:Neutrophils
2:Hepatocytes−1
3:Neural Progenitor Cells
4:Epithelial Cells−1
5:Fibroblasts
6:Neural Progenitor Cells
7:Muscle Cells
8:Muscle Cells
9:Hepatocytes−2
10:Kuffer
11:Cycling cells
12:Neurons
13:Melanocytes
14:Chondrocytes
15:Endothelial Cells
16:Erythrocytes
17:Epithelial Cells−2
18:Macrophages
19:Pancreatic Exocrine Cells
